# Identification of risk factors for 1-year mortality among critically ill older adults with hip fractures surgery: A single medical center retrospective study

**DOI:** 10.3389/fsurg.2022.973059

**Published:** 2022-08-31

**Authors:** Taijun Luo, Juxia Zhang, Haibin Zhou, Tao Xu, Wenchao Zhang, Geng Wang

**Affiliations:** Department of Anesthesiology, Beijing Jishuitan Hospital, Peking University Fourth School of Clinical Medicine, Beijing, China

**Keywords:** hip fracture surgery, mortality, NLR, arrhythmia, respiratory failure, older adults

## Abstract

**Aim:**

The purpose of this study was to analyze the potential risk factors for mortality 1 year after hip fracture surgery in critically ill older adults.

**Methods:**

We reviewed 591 critically ill older adults who underwent hip surgery at our institution from January 2018 to April 2021. We collected baseline demographics, clinical information, and 1-year survival status of the sample patients by means of medical record systems and follow-up phone calls. Patients were divided into survival and mortality groups based on survival within 1 year after surgery.

**Results:**

Based on the results of the 1-year postoperative follow-up of patients, we obtained 117 cases in the death group and 474 cases in the survival group, and this led to a 1-year mortality rate of 19.8% (117/591) after hip fracture in critically ill older adults at our hospital. The risk factors that influenced the 1-year postoperative mortality were identified as advanced age (HR:1.04, 95%, 1.01–1.06), preoperative arrhythmia (HR: 1.95, 95%, 1.26–2.70), high level of NLR (HR:1.03, 95%, 1.01–1.06), respiratory failure (HR: 2.63, 95%, 1.32–5.23), and acute cardiovascular failure. 5.23) and acute cardiovascular events (HR: 1.65, 95%, 1.05–2.59).

**Conclusion:**

Advanced age, preoperative arrhythmias, high levels of NLR, postoperative respiratory failure, and acute cardiovascular events were independent risk factors for survival of critically ill older adults with hip fracture at 1 year after surgery. Therefore, laboratory tests such as high levels of preoperative NLR can be an important indicator of patient prognosis.

## Introduction

With the increasing aging of society, the number of hip fractures in older adults continues to reach record highs, imposing a heavy medical and economic burden on the entire society (1). Studies have shown that surgery is the preferred method for hip fractures. In recent years, rapid improvements in the type of surgery, anesthesia methods, and perioperative management have provided surgical opportunities for critical ill older adults with hip fractures. However, it has also been found that the 1-year mortality rate after hip fracture surgery is as high as 20% or even 30% (2, 3). In this regard, many researchers have studied the factors influencing the risk of death after hip fracture in the general older adults, but there are fewer prognostic studies related to critically ill patients with hip fracture (4, 5). To fill this academic gap, the aim of this study is to analyze the incidence of 1-year mortality after hip fracture surgery in critically ill older adults. We retrospectively collected clinical data from all critically ill older hip fracture patients who underwent surgical treatment at Beijing Jishuitan Hospital from January 2018 to April 2021. Using the patients' 1-year postoperative survival rate as an indicator, we determined the factors affecting the patients' 1-year postoperative mortality.

## Methods

In 2018, our hospital established a joint management nursing ward for the older adults. The co-managed care team consists of orthopedic surgeons, emergency physicians, geriatricians, critical care physicians, anesthesiologists, nutritionists and physiotherapists who work together to provide diagnosis for both ordinary and critically ill older adults (6). Here, we defined patients who met the following criteria as critically ill older adults with hip fracture: (1) patients aged >85 years with multiple chronic diseases; (2) Patients with 2 or more comorbidities such as moderate to severe dementia, prolonged bed rest, cachexia, new myocardial infarction or cerebrovascular accident within 6 months, pulmonary disease combined with respiratory failure, cardiac arrhythmia with uncomfortable symptoms, stage 2–4 chronic renal insufficiency, chronic cardiac insufficiency NYHA ≥ II, severe anemia (Hb < 70 g/L) and other serious diseases; (3) Patients with unstable intraoperative conditions. For example, patients with unstable heart rate or blood pressure after admission to the operating room, high surgical blood loss, and significantly prolonged operative time. All critically ill older hip fracture patients who meet the above conditions should be transferred directly from the operating room to the ICU ward for observation after surgery.

This study involving human participants was reviewed and approved by the Institutional Review Board of Beijing Jishuitan Hospital. As this was a retrospective, anonymous data analysis, requirements regarding informed consent have been omitted here. All methods involved in the study were performed in accordance with relevant guidelines and protocols (7). Patients who underwent hip surgery at our hospital from January 2018 to April 2021 constituted our samples, whose inclusion criteria were (1) age ≥65 years; (2) fracture duration ≤21 days; (3) unilateral fracture; (4) low-energy injury; (5) critically ill older adults who had undergone hip surgery. Patient exclusion criteria were: (1) patients lacking relevant clinical information; (2) with old or pathological fractures; (3) high-energy injuries; (4) multiple trauma.

All data involved in the study were collected through electronic medical records and telephone follow-up. Through our electronic medical record system, we collected data on age, gender, BMI, ASA classification, co-morbidities, type of hip fracture, surgical waiting time, type of surgical operation, type of anesthesia, intraoperative adverse events, length of stay, preoperative creatinine, neutrophil/lymphocyte ratio (NLR), albumin, hemoglobin, c-reactive protein, NT- proBNP, perioperative period AKI [KDIGO guidelines diagnostic criteria (8)], delirium [diagnostic criteria (9)], pulmonary infection and circulatory complications. Also, during the study, the study investigators followed the patients by telephone 365 days after surgery.

Based on the follow-up results, patients were divided into survival and death groups. For continuous variables, the relevant data were expressed as mean ± standard deviation or median (interquartile range). For categorical variables, the relevant data were expressed as numbers (percentages). We compared all sample data between groups by using independent samples t-test or Mann-Whitney U-test for quantitative variables and chi-square test for categorical variables. In addition, the Kaplan-Meier method was used to perform analyses of patients' 1-year postoperative survival. Univariate and multivariate analyses were applied respectively in the analysis of independent risk factors for determining patients' mortality at 1 year after surgery. Of these, all variables with *P* < 0.05 in the univariate model were included in the multivariate model. Cox regression models were used for all patient survival analyses to compare outcomes between study groups and to identify predictors of patient postoperative mortality. The study was statistically analyzed using SPSS 24.0 software. All tests involved in the study were two-sided, and *P* < 0.05 was considered a statistically significant difference.

## Results

**Figure 1:**
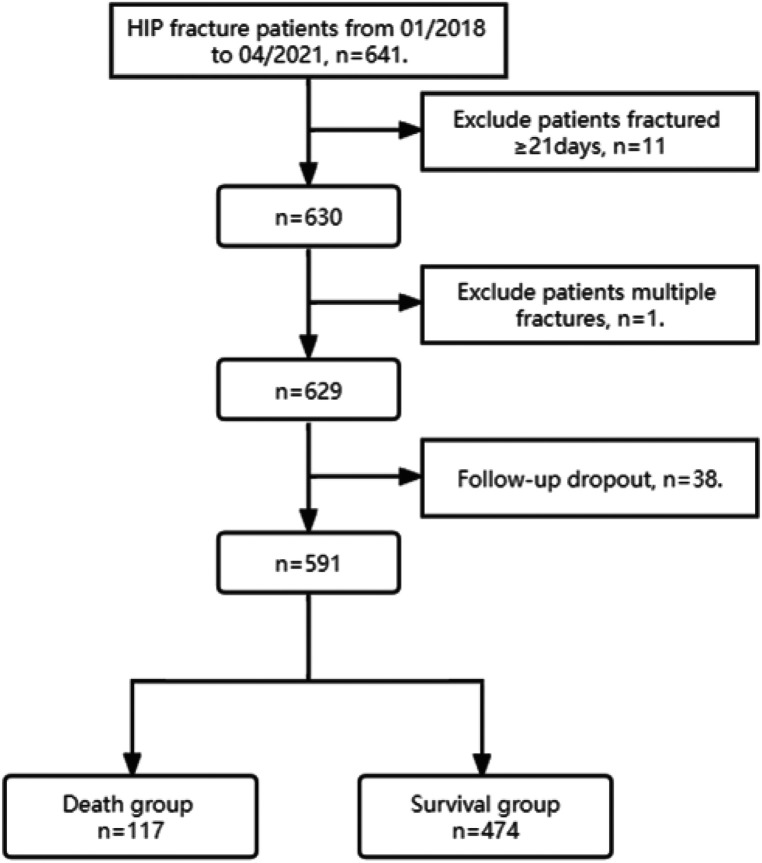
CONSORT diagram.

A total of 641 critically ill older adults with hip fractures were enrolled in this study from January 2018 to April 2021, and the final number of patients included in the study statistics was 591. 50 cases were excluded as they did not meet the criteria, including 11 cases of old fractures, 1 case of multiple fractures, and 38 cases lost to follow-up. The follow-up results showed 117 patients in the death group and 474 patients in the survival group, with a mortality rate of 19.8% (117/591) at 1 year postoperatively. (As shown in Figure 1).

The difference in age between the two groups was statistically significant (*P* < 0.05). Among them, preoperative chronic heart failure, arrhythmia, chronic kidney disease and Nottingham score were significantly higher in the death group than in the survival group (*P* < 0.05). In terms of laboratory tests, Scr, NLR, and NT-BNP were significantly higher in the death group than in the survival group (*P* < 0.05), and the use of general anesthesia was also significantly higher in the death group than in the survival group (*P* < 0.05) (see Table 1).

**Table 1:** Characteristics of hip fracture patients classified by survival status within 1 year.

	All (*n* = 591)	Death group (*n* = 117)	Survival group (*n* = 474)	*P*
Age (year)	86.0 (80.0,91.0)	88.0 (83.0,91.0)	85.0 (80.0,90.0)	0.006
Gender (M/F)	188/403	149/325	39/78	0.693
ASA (II/III/IV)	81/391/119	16/72/29	65/319/90	0.361
BMI	22.3 ± 4.1	21.5 ± 3.9	22.5 ± 4.1	0.013
Complications				
Hypertension	350 (59.2%)	64 (54.7%)	286 (60.3)	0.267
Diabetes	163 (27.6%)	32 (27.4%)	131 (27.6%)	0.950
CHD	200 (33.8%)	35 (29.9%)	165 (34.8%)	0.316
Arrhythmia^a^	151 (25.5%)	46 (39.3%)	105 (22.2%)	0.000
CHF	87 (14.7%)	25 (21.4%)	62 (13.1%)	0.023
Pulmonary disease	173 (29.3%)	45 (38.5%)	128 (27.0%)	0.015
CKD	162 (27.4%)	42 (35.9%)	120 (25.3%)	0.022
Anemia	121 (20.5%)	25 (21.4%)	96 (20.3%)	0.789
Alzheimer's	94 (15.9%)	19 (16.2%)	75 (15.8%)	0.912
Time to surgery (≥2 d)	567 (80.2%)	93 (81.1%)	381 (80.0%)	0.774
Type of fracture				
Intertrochanteric	352 (59.6%)	71 (60.7%)	281 (59.2%)	0.149
Femoral neck	224 (37.9%)	46 (39.3%)	178 (37.6%)	
Subtrochanteric	15 (2.5%)	0 (0%)	15 (3.2%)	
Type of anesthesia				
General	47 (8%)	3 (2.6%)	44 (9.3%)	0.016
Spinal	544 (92%)	114 (97.4%)	430 (90.7%)	
Laboratory examination				
Hemoglobin	111.3 ± 20.9	110.9 ± 21.2	111.9 ± 19.8	0.649
NT-proBNP	577.1 (272.6,1544.0)	918.0 (418.0,3183.5)	519.2 (248.7,1292.3)	0.000
NLR	9.8 ± 6.4	11.1 ± 7.0	9.5 ± 6.2	0.014
CRP	39.1 ± 50.0	39.1 ± 42.4	39.1 ± 51.7	0.999
SCr	65.0 (52.0,86.0)	71.0 (56.5,102,5)	64.0 (51.0,84.0)	0.024
Albumin	38.3 ± 4.5	37.9 ± 3.9	38.3 ± 4.6	0.299
Nottingham score	5.5 ± 0.9	5.6 ± 1.0	5.4 ± 0.9	0.026

ASA, American Society of Anesthesiologists; CHD, coronary heart disease; CHF, chronic heart failure; CKD, chronic kidney disease; NT-proBNP, NT-proB-type Natriuretic Peptide; NLR, neutrophil-lymphocyte ratio; CRP, C-reaction protein; SCr, serum creatinine.

^a^ Including atrial fibrillation, tachycardia, atrioventricular block and sick sinus node syndrome.

Patients in the death group had higher rates of postoperative respiratory failure and acute cardiovascular events than patients in the survival group (*P* < 0.05); the number of patients requiring ventilator and mask to assist with breathing was greater in the death group than in the survival group (*P* < 0.05); also, patients in the death group had significantly longer ICU stays than those in the survival group (*P* < 0.05) (see Table 2).

**Table 2:** Postoperative complications and ICU interventions.

	All (*n* = 591)	Death group (*n* = 117)	Survival group (*n* = 474)	*P*
Complications				
Pulmonary Infection	67 (11.3%)	17 (14.5%)	50 (10.5%)	0.224
Respiratory failure	22 (3.7%)	9 (7.7%)	13 (2.7%)	0.024
Acute cardiovascular events^a^	77 (13.0%)	25 (21.4%)	52 (11.%%)	0.003
AKI	24 (4.1%)	8 (6.8%)	16 (3.4%)	0.113
Delirium	20 (3.4%)	7 (6.0%)	13 (2.7%)	0.090
Length of hospital (d)	5.0 (4.0,7.0)	5.0 (4.0,7.0)	5.0 (4.0,7.0)	0.081
Length of ICU (d)	1.0 (1.0,2.0)	1.0 (1.0,3.0)	1.0 (1.0,2.0)	0.002
ICU interventions				
Ventilator ventilation	7 (1.2%)	4 (3.4%)	3 (0.6%)	0.031
Mask ventilation	18 (3%)	8 (6.8%)	10 (2.1%)	0.014
Antihypertensive	100 (16.9%)	23 (19.7%)	77 (16.2%)	0.378
Hypertensive	33 (5.6%)	6 (5.1%)	27 (5.7%)	0.811
Anti-arrhythmias	63 (10.7%)	18 (15.4%)	45 (9.5%)	0.064

^a^ Including acute coronary syndrome, acute exacerbation of chronic heart failure, malignant cardiac arrhythmia. AKI, acute renal insufficiency.

**Figure 2:**
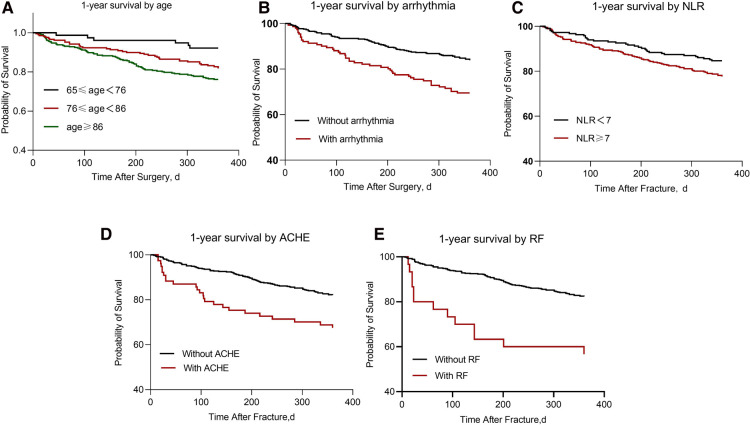
The graphs depict Kaplan-Meier estimated 1-year survival. (**A**) 1-year mortality by age (*P* < 0.000); (**B**) 1-year mortality by arrhythmias (*P* < 0.000); (**C**) 1-year mortality by NLR level (*P* = 0.04); (**D**) 1-year mortality by acute cardiovascular events (*P* < 0.000); (**E**) 1-year mortality by respiratory failure (*P* < 0.000). ACHE, acute cardiovascular events; RF, respiratory failure.

Kaplan-Meier survival curves are presented specifically in Figure 2, where Figure 2A clearly shows the lowest probability of survival for patients aged 86 years or older; in addition, from Figures 2B–E, we can see that patients without arrhythmias, acute cardiovascular events, respiratory failure, and low NLR levels have higher postoperative survival rates.

Cox regression analysis models showed that advanced age, preoperative comorbid arrhythmias, postoperative respiratory failure, and acute cardiovascular events were risk factors affecting 1-year postoperative survival in critically ill older adults with hip fractures (see Table 3).

**Table 3:** Predictive factors for 1-year mortality among hip fracture patients: multivariate analysis.

Variables	HR	95% CI	*P*
Age	1.04	1.01–1.06	0.011
Preoperative arrhythmias	1.95	1.26–2.70	0.002
Preoperative NLR	1.03	1.01–1.06	0.004
Respiratory failure	2.63	1.32–5.23	0.006
Acute cardiovascular events	1.65	1.05–2.59	0.029

## Discussion

In this observational, single-center, retrospective cohort study, the 1-year mortality and loss to follow-up rates were 19.8% (117/591) and 6.0% (38/629), respectively. Using multifactorial Cox proportional risk regression analysis, we found that advanced age, preoperative arrhythmias, high levels of NLR, postoperative respiratory failure, and acute cardiovascular events were independent risk factors for survival at 1 year after surgery.

First, advanced age has long been a consensus as an independent risk factor affecting postoperative survival of hip fracture in the older (10, 11). Cui et al. (12) reported the 1-year mortality rate of hip fracture in older adult in China from 2000 to 2018, and they found that the mortality rate of patients was positively correlated with age, in which the 1-year mortality rate of hip fracture patients over 90 years old exceeded 23.4%. Since older people usually suffer from more comorbidities as well as problems of organ function decline and cannot effectively resist perioperative surgical and anesthetic stimuli, effective elimination of adverse perioperative stimuli can improve postoperative survival in the older (13).

Second, older adults heart is often associated with pathological changes such as myocardial hypertrophy, fibrosis, inflammation, and persistent stiffness (14), and the sinoatrial node and conduction system are often affected by pathological changes, which manifest clinically as bradyarrhythmia, tachyarrhythmias, or tachycardia-bradycardia syndrome. Several studies have shown that bradyarrhythmia, tachyarrhythmias, or tachycardia-bradycardia syndrome are associated with higher postoperative mortality (15–17). Adunsky et al. (18) found that older adults with hip fractures combined with atrial fibrillation had a 1-fold increase in mortality at 1 year postoperatively (mean age 82.4 years) compared with patients without atrial fibrillation; Frenkel et al. (19) investigated 701 older hip fracture patients and showed a mean survival of 201 days in patients with atrial fibrillation and 377 days in patients without atrial fibrillation; in addition, Härstedt et al. (20) found that pathological bradycardia became an independent risk factor for medium- and long-term survival in older adults with hip fracture (mean age 82.6 years). Preoperative comorbid arrhythmias were found to be an independent risk factor for 1-year survival in critically ill older adults with hip fracture by our multivariate Cox proportional risk regression analysis. The 1-year mortality rate of hip fracture patients with combined arrhythmias was 1.95 times higher than that of patients without arrhythmias. Therefore, patients with arrhythmias should be evaluated preoperatively in a comprehensive manner to reduce the impact of adverse factors such as perioperative pain, anemia, and volume overload. Also, our study showed that postoperative acute cardiovascular events were associated with a 1.65-fold increase in 1-year mortality (95 CI, 1.05–2.59). Thus, comprehensive preoperative evaluation of patients with arrhythmias can also be effective in preventing the exacerbation or worsening of arrhythmias and reduce the incidence of acute postoperative cardiovascular events.

The occurrence of perioperative respiratory failure is also an important risk factor affecting the prognosis of hip fracture in older adults (age >65 years) (4). The main causes of respiratory failure in older adults are the following: First, with age, degenerative physiological changes occur in lung compliance, respiratory mucosa, cilia movement, lung volume, and respiratory rate in older adults (21). With the gradual decline of systemic immunity, the respiratory tract is susceptible to bacterial and viral attack, leading to lung infection and, in serious cases, respiratory failure; Second, stimuli such as trauma and pain can cause a state of systemic stress, leading to increased oxygen consumption and increased work done by respiratory muscles, which may develop into respiratory muscle weakness and respiratory failure (22); Finally, respiratory failure can also occur in older adults with pre-existing conditions in response to traumatic stimuli. Chen et al. (23) found that older hip fracture patients (67–83 years) with hypertension, obstructive lung disease, bronchiectasis, and a history of respiratory failure were significantly more likely to develop respiratory failure postoperatively. Wang et al. (24) followed 144 patients (90–97 years) with intertrochanteric fractures and showed that the development of postoperative respiratory failure was an independent risk factor for survival for 1–2 years after surgery. In addition, our study also found that the occurrence of respiratory failure in the perioperative period was an important risk factor for survival at 1 year after surgery. Patients who developed respiratory failure had a 1.95-fold higher mortality rate at 1 year than those who did not develop respiratory failure (95 CI, 1.26–2.70). Therefore, reducing the occurrence of perioperative respiratory failure is beneficial in reducing postoperative mortality.

Recent studies have shown NLR to be a potential marker for predicting mortality in older adults in different clinical settings (25, 26). Vaughan-Shaw et al. (27) found NLR to be a good predictor of 30-day mortality, 6-month mortality, and 1-year mortality after emergency abdominal surgery in the older adults(80–95 years). Forget et al. (28) showed that NLR on postoperative day 5 was a risk factor for postoperative mortality and cardiovascular complications in older hip fracture patients (mean age 85 years, age range: 66–102). In a study, Chen et al (29) found that higher preoperative and postoperative NLR was associated with a higher risk of long-term mortality after hip surgery in older patients equal to or more than 65 years of age. Our findings also suggest that high preoperative NLR increases mortality within 1 year postoperatively. Therefore, we need to pay more attention to the prevention of acute adverse events in patients with high perioperative NLR levels. The reduction of NLR has an important impact in reducing inflammatory status and improving immune responsiveness in critically ill older hip fracture patients. Therefore, clinicians can individualize patient interventions, such as preoperative analgesia, statins, or aspirin, depending on NLR levels.

In reviewing the entire study, we still have many limitations. First, although we performed postoperative follow-up with our patients, many families kept the cause of postoperative death confidential or could not describe it accurately, preventing us from conducting an in-depth summary and analysis of the causes of postoperative death; second, as a single-center retrospective study with a moderate sample size, it was not yet possible to adequately compare the effects of anesthesia type and ICU intervention on postoperative survival time in critically ill older hip fracture patients.

## Data Availability

The original contributions presented in the study are included in the article/Supplementary Materials, further inquiries can be directed to the corresponding author/s.
